# Influence of three BALB/c substrain backgrounds on the skin tumor induction efficacy to DMBA and TPA cotreatment

**DOI:** 10.1186/s42826-020-00063-z

**Published:** 2020-09-03

**Authors:** Mi Ju Kang, Jeong Eun Gong, Ji Eun Kim, Hyeon Jun Choi, Su Ji Bae, Yun Ju Choi, Su Jin Lee, Min-Soo Seo, Kil Soo Kim, Young-Suk Jung, Joon-Yong Cho, Yong Lim, Dae Youn Hwang

**Affiliations:** 1 Department of Biomaterials Science, College of Natural Resources and Life Science/Life and Industry Convergence Research Institute/Laboratory Animals Resources Center, Pusan National University, Miryang, South Korea; 2 Laboratory Animal Center, Daegu-Gyeongbuk Medical Innovation Foundation, Daegu, South Korea; 3 College of Veterinary Medicine, Kyungpook National University, Daegu, South Korea; 4 College of Pharmacy, Pusan National University, Busan, South Korea; 5 Exercise Biochemistry Laboratory, Korea National Sport University, Seoul, South Korea; 6 Department of Clinical Laboratory Science, College of Nursing and Healthcare Science, Dong-Eui University, Busan, South Korea

**Keywords:** BALB/c, BALB/cKorl, Substrains, DMBA+TPA, Cisplatin, Skin tumor

## Abstract

Differences in responsiveness of BALB/c substrains have been investigated in various fields, including diabetes induction, corpus callosum deficiency, virus-induced demyelinating disease, aggressive behavior and osteonecrosis. However, induction efficacy of skin tumor remains untried. We therefore investigated the influence of BALB/c substrain backgrounds on the skin tumor induction efficacy in response to DMBA (7,12-Dimethylbenz[a]anthracene) and TPA (12-O-tetradecanoylphorbol-13-acetate) cotreatment. Alterations in the levels of tumor growth related factors, histopathological structure, and the expression to tumor related proteins were measured in three BALB/c substrains (BALB/cKorl, BALB/cA and BALB/cB) after exposure to DMBA (25 μg/kg) and three different doses of TPA (2, 4 and 8 μg/kg). The average number and induction efficacy of tumors in response to DMBA+TPA treatment were significantly greater in the BALB/cKorl substrain than in BALB/cA and BALB/cB. However, cotreatment with DMBA+TPA induced similar responses for body and organ weights of all three substrains. Few differences were detected in the serum analyzing factors, while similar responsiveness was observed for blood analyzing factors after DMBA+TPA treatment. Furthermore, the three BALB/c substrains exhibited similar patterns in their histopathological structure in DMBA+TPA-induced tumors. The expression levels of apoptotic proteins and tumor related proteins were constantly maintained in all three BALB/c substrains treated with DMBA+TPA. In addition, the responsiveness to cisplatin treatment was overall very similar in the three BALB/c substrains with DMBA+TPA-induced tumors. Taken together, these results indicate that genetic background of the three BALB/c substrains does not have a major effect on the DMBA+TPA-induced skin carcinogenesis and therapeutic responsiveness of cisplatin, except induction efficacy.

## Introduction

BALB/c mice are amongst the most widely used inbred strains in cancer and immunology studies [1]. These mice are derived from the Bagg albino strain provided by Halsey J. Bagg of the Memorial Hospital (NY, USA), which was obtained from a mouse dealer in Ohio in 1913. In 1935, the animals were transferred with George Davis Snell to The Jackson Laboratory and established as the stable BALB/cJ strain [2, 3]. Thereafter, in 1935, the highly reproductive and less aggressive BALB/cByJ mice were separated from the BALB/cJ strain. A third substrain (BALB/c AnNCrl) was separated from BALB/c and BALB/cByJ mice between the fifties to seventies. These three substrains have divergent genetic and behavioral differences and reproducibility [4]. Based on above phylogenetic trees, the correlation between genetic differences and aggressive behavior of BALB/c mice has received great attention from researches [2, 4].

Influences of BALB/c substrain characteristics were evaluated in various phenotypical responsiveness. Different phenotypes of BALB/cJ and BALB/cByJ mice are involved in the genetic and environmental control of diabetes induced by multi-dose streptozotocine [5]. A variation in the deficiency of the corpus callosum was observed in 13 seperated lines established by full-sib inbreeding BALB/c mice, while a spontaneous change occurred only in the BALB/cWah 1 line after 7 generation of inbreeding [6]. Susceptibility to Theiler’s murine encephalomyelitis virus (TMEV)-induced demyelinating disease analyzed in BALB/c substrains revealed that the BALB/cJ and BALB/cAnNCr mice were susceptible, whereas the BALB/cByJ and BALB/cCum strains were observed to be resistant [7]. Furthermore, significant differences were determined for aggressive behaviors identified in the BALB/cJ, BALB/cByJ and BALB/cAnNCr substrains [2, 4]. BALB/cJ mice were more susceptible to dexamethasone-induced osteonecrosis compared to BALB/cAnNHsd mice [8]. However, no studies provide any scientific evidence for differences in BALB/c substrains with respect to skin tumor induction efficacy of DMBA+TPA, and the therapeutic responsiveness of cisplatin.

In order to investigate the influence of BALB/c susbstrain backgrounds on two-stage skin carcinogenesis, this study therefore compared the skin tumor induction efficacy in the DMBA+TPA model, as well as evaluated anti-tumor effects in the DMBA+TPA model treated with cisplatin, by assessing the effects in BALB/cKorl stock and two commercial stocks (BALB/cA and BALB/cB).

## Materials and methods

### Care and management of animals

All animal protocols used in this study were reviewed and approved by the Pusan National University-Institutional Animal Care and Use Committee (PNU-IACUC, approval number PNU-2018-1955). Female BALB/c (7-weeks-old) were obtained from three different sources. The BALB/cKorl mice were kindly provided by the Department of Laboratory Animal Resources of the National Institute of Food and Drug Safety Evaluation (NIFDS, Chungju, Korea). The other two strains (BALB/cA and BALB/cB) were purchased from vendors located in the United States (Vendor A) and Japan (Vendor B), respectively. All BALB/c mice were maintained and treated at the Laboratory Animal Resource Center of Pusan National University, which is certified by the Korea Food and Drug Administration (KFDA, Accredited Unit Number 000231), and Association for Assessment and Accreditation of Laboratory Animal Care (AAALAC) International (Accredited Unit Number; 001525). During the entire experimental period, all mice were maintained in a specific pathogen-free (SPF) state under a strict light cycle (lights on at 08:00 h and off at 20:00 h), at 23 ± 2°C and 50 ± 10% relative humidity. Animals were provided with *ad libitum* access to a standard irradiated chow diet (Samtako BioKorea Inc., Osan, Korea).

### Design of animal experiment

**Fig. 1:**
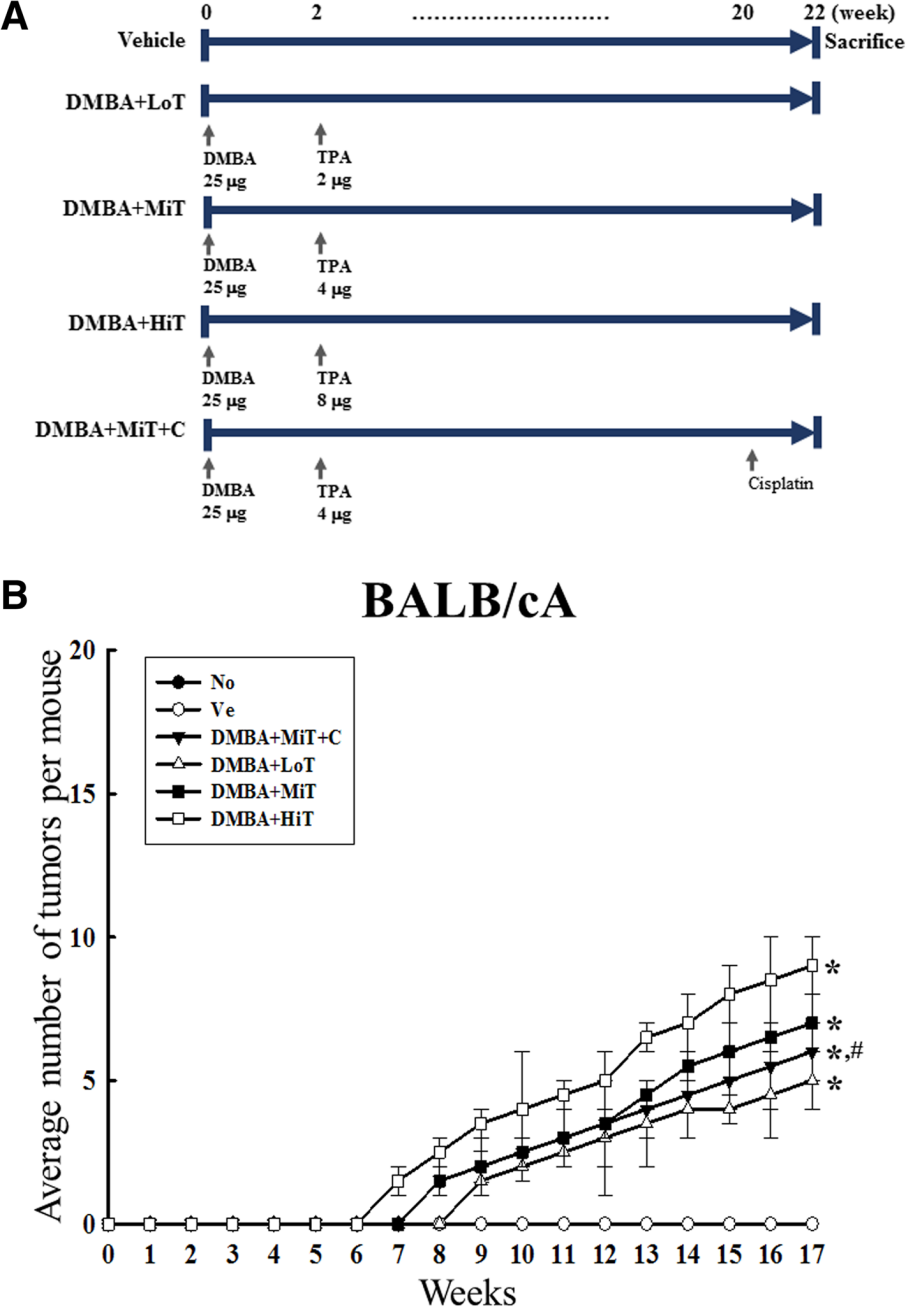
Experimental scheme and average tumor number in the three BALB/c substrains. **a** After applying single dose DMBA, three different doses of TPA were applied to the shaved back skin, twice weekly for 20 weeks. Also, one group of BALB/c mice was administered DMBA and medium dose TPA, with subsequent intraperitoneal injection of cisplatin as anti-tumor drug. **b** Average number of tumors. Only papilloma with tumor size 2 cm or more were counted, and are represented in the graph. Data represent the mean ± SD. *, *p* < 0.05 compared to the No treated group. #, *p* < 0.05 compared to the Vehicle treated group

BALB/c mice of each substrain (*n* = 48, 9-weeks-old) were assigned to one of the three groups: No treated group (*n* = 8), Vehicle treated group (*n* = 8) or DMBA treated groups (*n* = 32). On day 1, all mice in the DMBA treated group were shaved, followed by a single topical application of DMBA (Sigma-Aldrich Co., St. Louis, MO, USA; 25 μg in 200 ml acetone), whereas mice in the Vehicle group were treated with only acetone solution. Following administration of DMBA, the treated mice were further classified into four groups: DMBA+Low dose TPA treated group (DMBA+LoT treated group, *n* = 8), DMBA+Medium dose TPA treated group (DMBA+MiT treated group, *n* = 8), DMBA+High dose TPA treated group (DMBA+HiT treated group, *n* = 8), and DMBA+Medium dose TPA + Cisplatin treated group (DMBA+MiT + C treated group, *n* = 8). The DMBA+LoT, DMBA+MiT and DMBA+HiT groups received twice weekly topical applications of three different doses (2, 4 and 8 μg/kg, respectively) of TPA (Sigma-Aldrich Co.; 200 ml of 10^− 4^ M solution in acetone) for 22 weeks (Fig. 1a). The DMBA+MiT + C treated group was treated the same as the DMBA+MiT treated group, with an additional intraperitoneal injection of cisplatin (Sigma-Aldrich Co., 1 mg/kg), every 2 days for the last 2 weeks. After the final treatment, all animals were sacrificed using CO_2_ gas. Blood, tumor and tissue samples were subsequently collected and stored in Eppendorf tubes at -70 °C until further assays.

### Measurement of tumor number and incidence

After measuring the tumor width on the back skin of mice using external calipers (Matusutoyo, Tokyo, Japan), the number of tumors having diameter more than 2 cm was counted. The average number of tumors per mice was calculated based on the total number of tumors obtained. Moreover, tumor incidence was calculated using the following formula:

$$ \mathrm{Tumor}\ \mathrm{incidence}\ \left(\%\right)=\mathrm{A}/\mathrm{B}\times 100 $$

where A is the number of animals with tumor, and B is the total number of experimental animals in each group.

### Measurement of body and organ weights

Throughout the experimental period, body weights of mice in all subset groups were measured daily at 10:00 am using an electronic balance (Mettler Toledo, Greifensee, Switzerland), according to the KFDA guidelines. In addition, the weights of five organs harvested (liver, lung, kidney, thymus and spleen) from the sacrificed BALB/c mice were determined using the same method employed to measure body weight.

### Whole blood and serum analysis

After 22 weeks of the experimental process, all mice were fasted for 8 h, following which anesthesia was induced by intraperitoneal injection of Alfaxan (JUROX Pty Limited, Rutherford, Australia, 13 mg/kg body weight i.v.), and blood was subsequently collected from the abdominal veins using a 1 ml syringe attached to a needle (21 SWG) [9, 10]. Mice were then sacrificed by cervical dislocation. Blood analysis and serum biochemistry were performed for all collected samples. Whole blood was placed in plain capped bottles containing ethylenediaminetetraacetate (EDTA), and the components were analyzed using an automated cell counter (Beckman-Coulter Inc., Miami, Florida) with standard calibration, according to the manufacturer’s instructions. The levels of white blood cells (WBC), red blood cells (RBC), hemoglobin (HGB), hematocrit (HCT), mean corpuscular volume (MCV), mean corpuscular hemoglobin (MCH), mean corpuscular hemoglobin concentration (MCHC), corpuscular hemoglobin concentration mean (CHCM), corpuscular hemoglobin content (CH), hemoglobin concentration distribution width (HDW), platelets (PLT), and mean platelet volume (MPV) were measured in duplicate for each sample.

Serum was obtained for biochemical analysis by centrifuging the whole blood at 1,500 xg for 10 min. Serum biochemical components, including alkaline phosphatase (ALP), alanine aminotransferase (ALT), aspartate aminotransferase (AST), calcium (Ca) and low density lipoprotein (LDH), were assayed using an automatic serum analyzer (Hitachi 747; Hitachi, Tokyo, Japan). All assays were measured in duplicate using fresh serum.

### Histological analysis

Skin and tumor tissues harvested from mice of all subset groups were fixed overnight in 10% neutral buffered formaldehyde (pH 6.8). The dehydrated tissues were subsequently embedded in paraffin wax, followed by a series of skin and tumor sections (4 μm) cut from the paraffin-embedded tissues using a Leica microtome (Leica Microsystems, Bannockburn, IL, USA). The sections were then deparaffinized with xylene (Daejung, Gyeonggi-do, Korea), rehydrated with graded ethanol (decreasing concentrations of 100–70%), and finally washed with distilled water. The tissue sections on slides were stained with hematoxylin (Sigma-Aldrich Co.) and eosin (Sigma-Aldrich Co.), and washed with dH_2_O. Alterations in the histological structure of tumors were observed under the Leica Application Suite (Leica Microsystems). The tumor type and pathological features were characterized by a pathologist, Dr. Sang Gu Lee, at DDPartner Co. (Seoul, Korea).

### Western blot

Total proteins prepared from the tumor tissue were separated by 4–20% sodium dodecyl sulfate-polyacrylamide gel electrophoresis (SDS-PAGE) for 2 h, after which the resolved proteins were transferred to nitrocellulose membranes for 2 h at 40 V. Each membrane was then incubated separately, overnight at 4°C, with the following primary antibodies: p53 (Sigma-Aldrich Co.), p27 (Sigma-Aldrich Co.), Bax (Abcam, Cambridge, UK), anti-Bcl2 (Abcam), caspase-3 (Cell Signaling Technology, Danvers, MA, USA), and anti-β-actin (Cell Signaling Technology). The membranes were then washed with washing buffer (137 mM NaCl, 2.7 mM KCl, 10 mM Na_2_HPO4, and 0.05% Tween 20) and incubated with HRP-conjugated goat anti-rabbit IgG antibody (Invitrogen) at a 1:1000 dilution, at room temperature for 1 h. Membrane blots were developed using Amersham ECL Select Western Blotting detection reagent (GE Healthcare).

### Statistical analysis

Statistical analyses were performed with SPSS for Windows, release 10.10, standard version (SPSS, Inc., Chicago, IL, USA). One-way analysis of variance (ANOVA) followed by Tukey’s post hoc test for multiple comparisons was performed to identify significant differences between groups. All values are reported as the mean ± S.E.M, and a *p*-value (*p* < 0.05) is considered as significant.

## Result

### Influence of BALB/c substrain background on the tumor induction efficacy in response to DMBA+TPA cotreatment

**Fig. 2:**
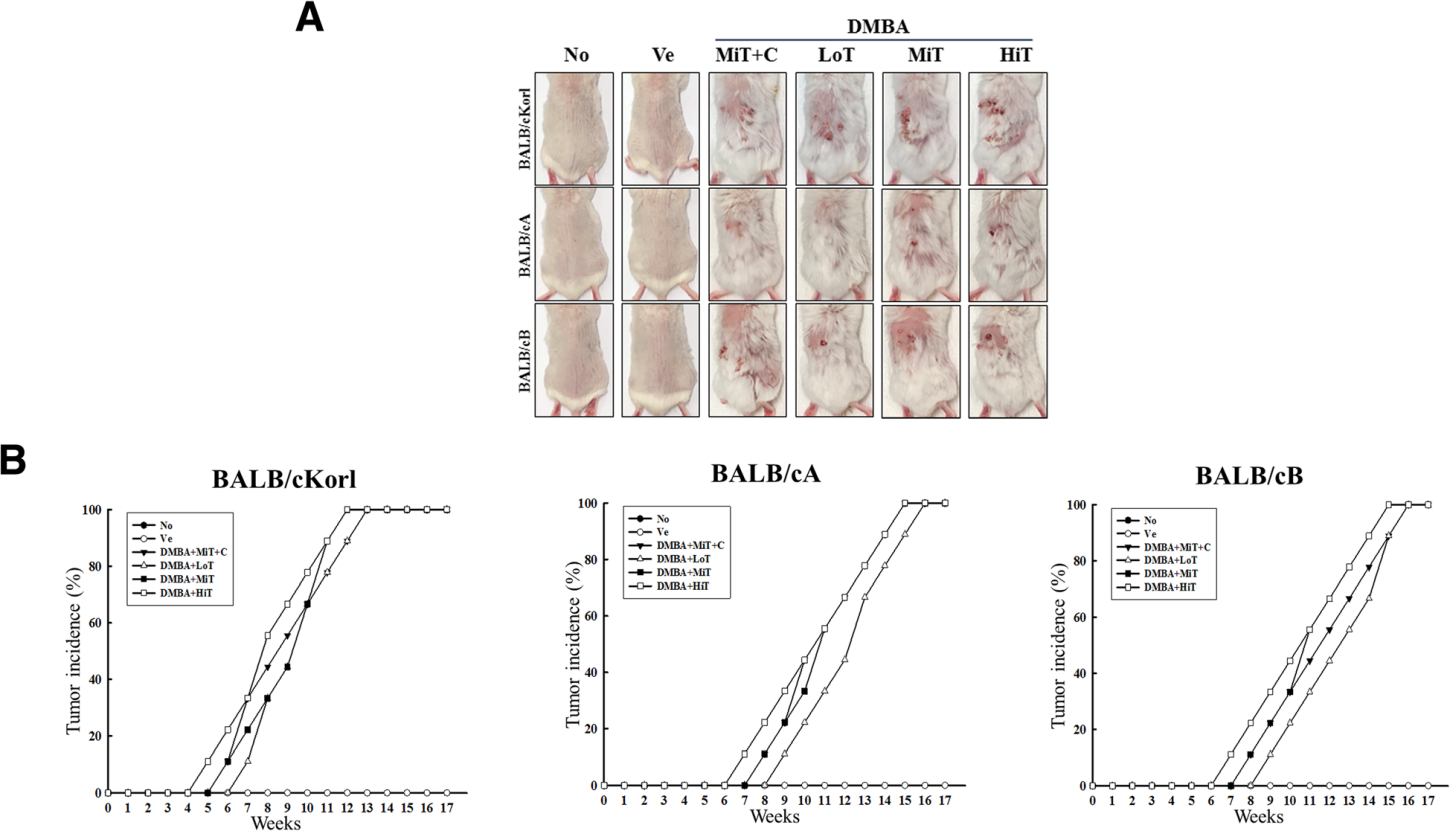
Tumor growth and incidence in three BALB/c substrains after DMBA and TPA cotreatment. **a** Morphological images of the tumor developed on the back skin of three BALB/c substrains treated with DMBA and TPA at 22 weeks. **b** Tumor incidence calculated as described in Materials and Method

In order to investigate the influence of the background of BALB/c substrains on tumor induction efficacy to DMBA+TPA exposure, the average number, morphology and incidence rate of tumors were measured after stimulating with three different concentrations of TPA in DMBA sensitized mice. The formation of small solid tumors was observed on the back skin of all BALB/c substrains after treatment with DMBA+TPA. As shown in Fig. 1b, tumor growth was over a prolonged duration in all three BALB/c substrains (measurement beginning from day 3), and the average increase in tumor numbers was dependent on the concentration of TPA. However, the tumor increase rates between groups differed, and was greater in BALB/cKorl than in BALB/cA and BALB/cB. Especially, the average tumor numbers rapidly increased in the BALB/cKorl group, as compared to the BALB/cA and BALB/cB groups. In the cisplatin treatment group, a significant decrease of tumor number was observed in only BALB/cKorl and BALB/cA, while constant numbers were maintained in BALB/cB mice (Fig. 1b). Furthermore, visual observations for tumor number on the back skin revealed a similar pattern to the increase curve (Fig. 2a). In addition, tumor induction rate calculated from the specified formula showed a tendency similar to the visual observation and average number of tumors. Tumor incidence in BALB/cKorl mice reached 100% at 12 weeks, while the same levels were detected in BALB/cA and BALB/cB at 15 weeks post DMBA treatment. The DMBA+MiT + C treated group showed a similar pattern for tumor incidence, when compared to the three BALB/c substrains (Fig. 2b). These results indicate that background of the three BALB/c substrains affects the skin tumor induction efficacy in response to DMBA+TPA exposure. Especially, the BALB/cKorl substrain shows better sensitivity to DMBA+TPA skin carcinogenesis than the other substrains.

### Influence of BALB/c substrain background on alterations in body and organ weight after exposure to DMBA+TPA cotreatment

**Fig. 3:**
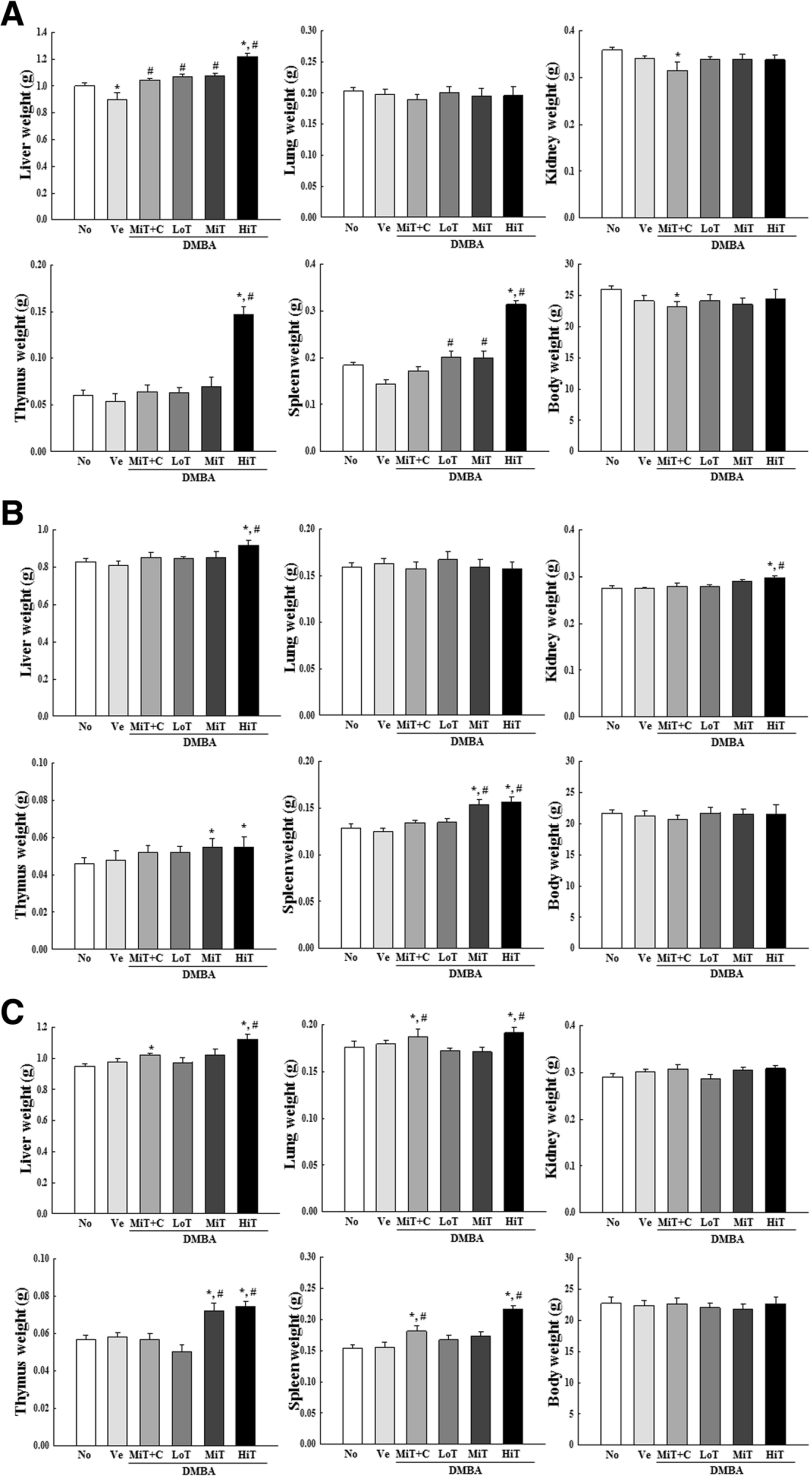
Body and organ weight of three BALB/c substrains after DMBA and TPA cotreatment. After final treatment in **a** BALB/cKorl, **b** BALB/cA and **c** BALB/cB, five internal organs, including liver, lung, kidney, thymus and spleen, were collected from mice of subset groups, and their weights were measured in duplicate. Data represent the mean ± SD. *, *p* < 0.05 compared to the No treated group. #, *p* < 0.05 compared to the Vehicle treated group

We investigated the influence of BALB/c substrain background on alterations in the body and organ weights in the three BALB/c substrains exposed to DMBA+TPA. To achieve this, the weights of body, liver, lung, kidney, thymus and spleen were measured after applications of three different concentrations of TPA in DMBA sensitized mice. Most organs showed similar alteration patterns in weight in all three substrains, although few differences were observed. Especially, a significant increase was observed in the weights of the thymus and spleen in the DMBA+HiT treated group, as compared with other groups. These patterns were maintained in all BALB/c substrains. Furthermore, cisplatin treatment induced a slight decrease in body and kidney weights of the BALB/cKorl substrain, increased liver weight in the BALB/cKorl and BALB/cB substrains, and enhanced lung weight in the BALB/cB substrain (Fig. 3). These results indicate that background of the three BALB/c substrains does not affect major alterations in the body and organ weights of mice after treatment with DMBA and TPA.

### Influence of BALB/c substrain background on serum levels and blood analyzing factors in response to DMBA+TPA cotreatment

**Fig. 4:**
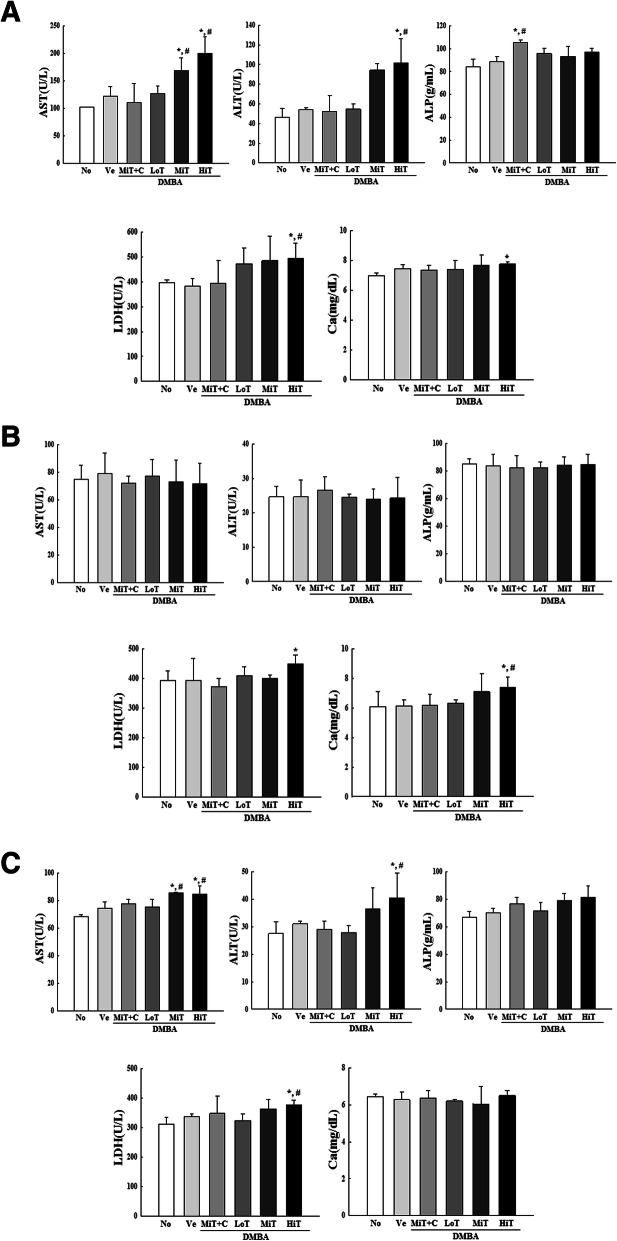
Serum parameters of three BALB/c substrains after DMBA and TPA cotreatment. After final treatment in **a** BALB/cKorl, **b** BALB/cA and **c** BALB/cB, serum levels of four parameters (AST, ALT, ALP, LDH) and Ca were measured, as described in Materials and Methods. Data represent the mean ± SD. *, *p* < 0.05 compared to the No treated group. #, *p* < 0.05 compared to the Vehicle treated group

To examine the influence of the background of BALB/c substrains on serum levels and blood analyzing factors in the three BALB/c substrains treated with DBMA+TPA, alterations in the concentrations of serum and blood analyzing factors were measured after stimulating with three different concentrations of TPA in DMBA sensitized mice. Among the serum analyzing factors, a significant difference between substrains was observed in the concentrations of AST, ALT and Ca. The concentration of AST and ALT were dose-dependently increased due to TPA exposure in the BALB/cKorl and BALB/cB substrains, but maintained constant levels in the BALB/cA substrain. However, a reverse pattern was detected for Ca concentration (Fig. 4). Blood analysis revealed that the concentrations of 12 factors were maintained constant in all DMBA+TPA treated groups as compared to Vehicle treated group. Furthermore, these patterns were similarly detected in BALB/cKorl, BALB/cA and BALB/cB substrains (Data not shown). These results indicate that the background of the three BALB/c substrains does not affect major alterations in serum and blood analyzing factors after exposure to DMBA and TPA.

### Influence of BALB/c substrain background on the histopathological structure of skin and tumor in response to DMBA+TPA cotreatment

**Fig. 5:**
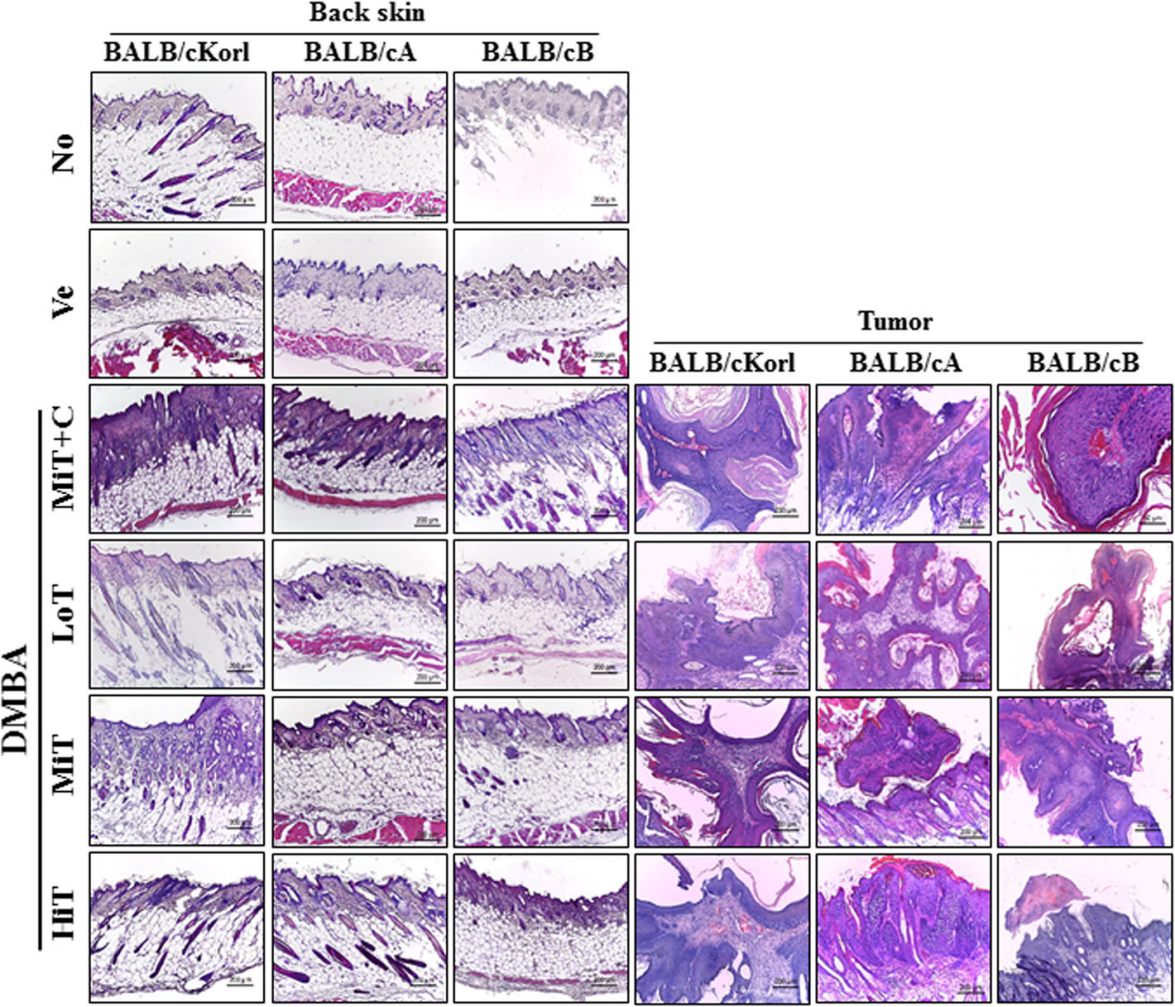
Histopathological analysis of skin and tumor tissue. After harvesting the skin and tumors from DMBA+TPA treated BALB/c substrains, the histopathological changes in slide sections of skin (left column) and tumor tissue (right column) were identified by staining with hematoxylin and eosin and observation at 200× magnification

We further examined the influence of BALB/c substrain background on the histopathological structure of skin and tumor in the three BALB/c substrains treated with DBMA+TPA for 22 weeks. We examined for changes in the histological structure of back skin and tumor after exposure to three different concentrations of TPA in DMBA sensitized mice. Examination of skin tissue harvested from the back of mice revealed significantly increased thickness of the epidermis and dermis in all DMBA+TPA treated groups, as compared with Vehicle treated group. Moreover, a remarkable accumulation of immune cells and enhanced number of hair follicles were detected in the dermis and hypodermis of DMBA+TPA treated groups. These patterns were similarly detected in all the three BALB/c substrains (Fig. 5). Tumor tissue assessment commonly detected squamous cell carcinoma, with alterations of nuclear size, shape, margin, chromatin pattern, nucleoli, and perinucleolar space, in the three substrains. No significant differences were observed in the three substrains, although tumor severity revealed a TPA dose-dependent pattern (Fig. 5). Furthermore, tumor progression was significantly suppressed with cisplatin treatment in the DMBA+MiT treated model. These suppressions were constantly maintained in all three BALB/c substrains (Fig. 5). Taken together, these results indicate that background of the three BALB/c substrains does not majorly affect alterations in the histopathological structure in the DMBA+TPA induced tumors of BALB/cKorl, BALB/cA and BALB/cB substrains.

### Influence of BALB/c substrain background on the regulation of tumor suppressor and apoptotic proteins in tumors induced in response to DMBA and TPA cotreatment

**Fig. 6:**
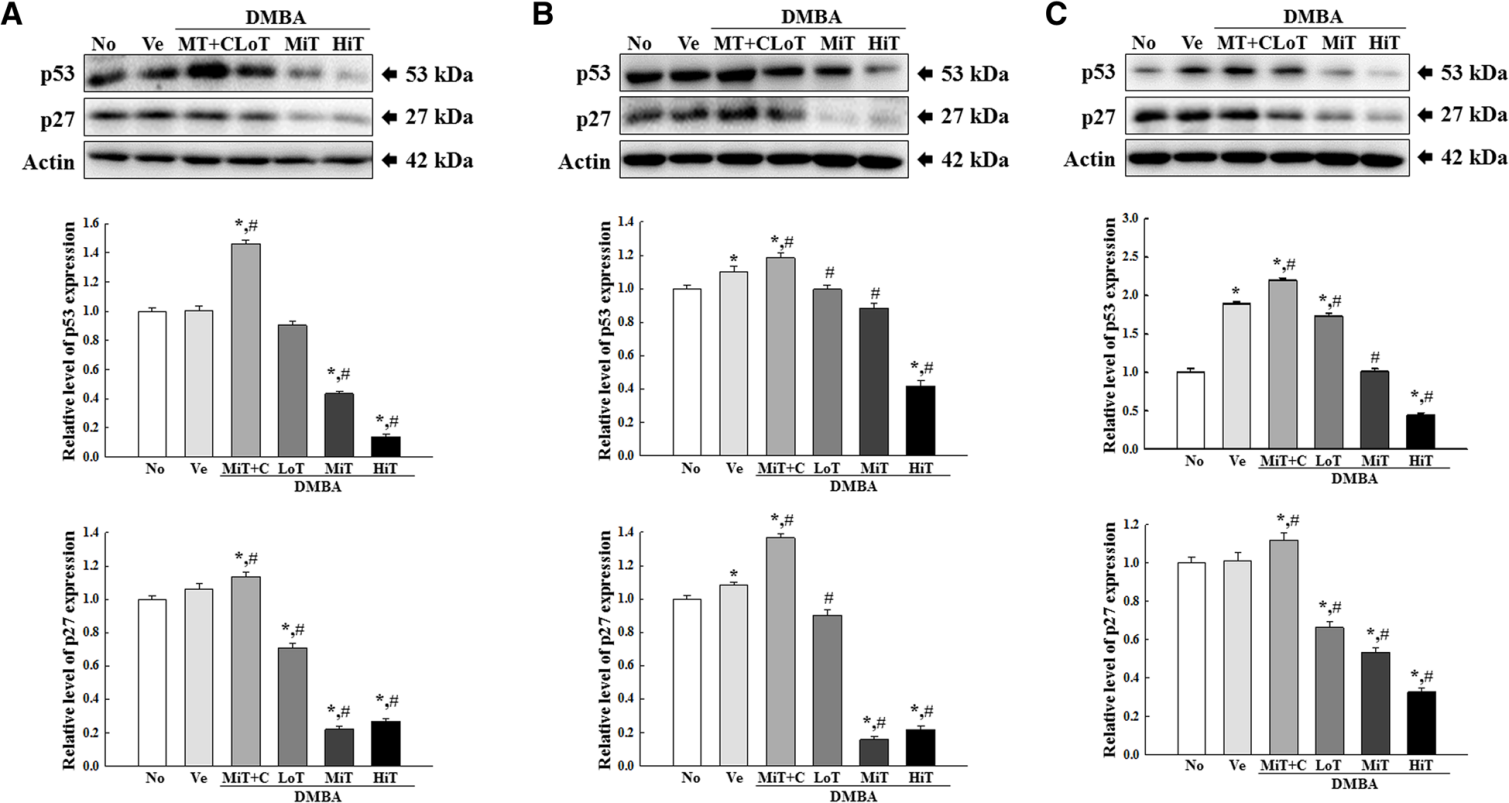
Protein expressions of p27 and p53 in tumor samples from the DMBA+TPA treated **a** BALB/cKorl, **b** BALB/cA, and **c** BALB/cB substrains. Expression levels of p53 and p27 proteins were determined by Western blot analysis using HRP-conjugated anti-rabbit IgG antibodies. Band intensity of each protein was measured using an imaging densitometer, and expressions of the two proteins were calculated relative to the intensity of β-actin protein. Data represent the mean ± SD. *, *p* < 0.05 compared to the No treated group. #, *p* < 0.05 compared to the Vehicle treated group

**Fig. 7:**
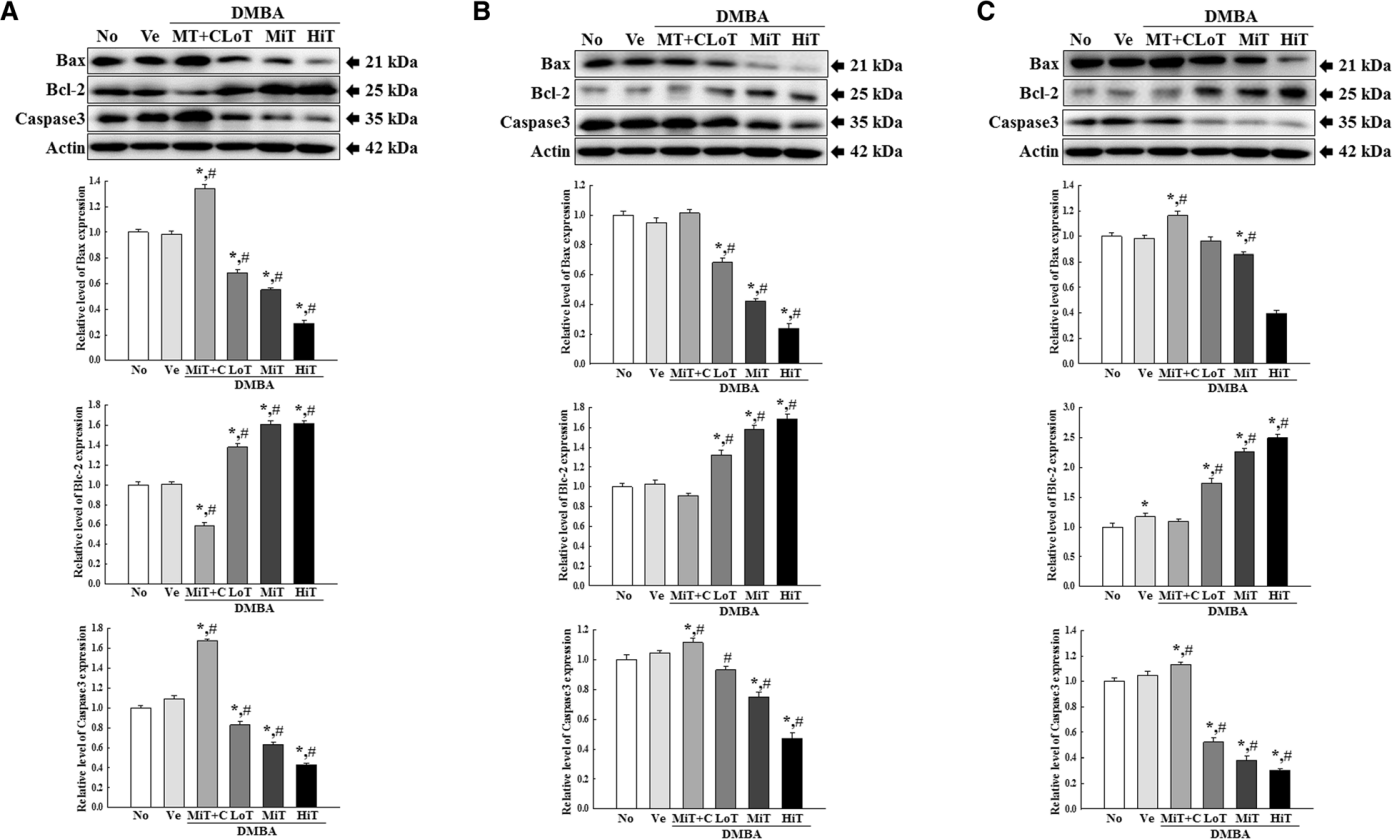
Protein expressions of Bax, Bcl-2 and Caspase-3 in tumor samples from the DMBA+TPA treated **a** BALB/cKorl, **b** BALB/cA and **c** BALB/cB substrains. Expression levels of Bax, Bcl-2 and Caspase-3 proteins were determined by Western blot analysis using HRP-conjugated anti-rabbit IgG antibodies. Band intensity of each protein was measured using an imaging densitometer, and expressions of the three proteins were calculated relative to the intensity of β-actin protein. Data represent the mean ± SD. *, *p* < 0.05 compared to the No treated group. #, *p* < 0.05 compared to the Vehicle treated group

Next, we examined the influence of BALB/c substrain background on the regulation of tumor suppressors and apoptotic proteins during the two-stage skin carcinogenesis in the three BALB/c substrains. To achieve this, alterations in the expression levels of p53, p27, Bax, caspase-3 and Bcl-2 proteins were measured in tumors harvested from mice of the three substrains exposed to DMBA+TPA for 22 weeks. A similar alteration pattern was observed for the expressions of all proteins in the DMBA+TPA treated groups. The expression levels of p53 and p27 proteins decreased in a TPA dose-dependent manner in the DMBA sensitized mice. These decrease patterns were similar for BALB/cKorl, BALB/cA and BALB/cB substrains (Fig. 6). Furthermore, expression levels of Bax and caspase-3 were significantly and dose-dependently decreased in the DMBA+LoT, DMBA+MiT and DMBA+HiT treated groups, as compared to the Vehicle treated group. However, the expression of Bcl-2 was markably enhanced in the same groups. These alteration patterns were constantly maintained in the BALB/cKorl, BALB/cA and BALB/cB substrains (Fig. 7). A similar regulation pattern was observed in all DMBA+TPA treated BALB/c substrains after exposure to cisplatin (Fig. 7). The above results indicate that background of the three BALB/c substrains does not affect major alterations in the expression levels of apoptotic proteins and tumor related proteins in BALB/cKorl, BALB/cA and BALB/cB substrains, after treatment with DMBA and TPA.

## Discussion

Korl code has been established as one of the novel substrains of several inbred and outbred mice, including C57BL/5, ICR, BALB/c and DBA/2, at the National Institute of Food and Drug Safety Evaluation (NIFDS), Korea [11, 12]. Until now, the biological phenotypes of Korl (Korl:ICR, C57BL/6NKorl, BALB/cKorl and DBA/2Korl) were compared with other substrains provided by commercial venders, to verify the stability of their characteristics. These phenotypes showed similar responses to various disease-inducing agents in cancer biology, neurobiology, immunology and microbiology [13–16]. As part of the current study, we investigated the influence of BALB/cKorl substrain background on DMBA+TPA induced skin carcinogenesis. To achieve this, alterations in the skin tumor induction efficacy, histopathological structure, and tumor-related protein expressions were compared in BALB/cKorl, BALB/cA and BALB/c substrains. Although the BALB/cKorl substrain showed no significant differences when compared to two BALB/c substrains (the most common biological phenotypes available) procured from commercial vendors, the BALB/cKorl substrain showed a higher level of tumor incidence.

The two-stage protocol for skin tumorigenesis was first established in the 1920s, when investigating the appearance of tumors in mouse skin treated with carcinogenic tar [17]. This protocol was used for investigating the therapeutic effect of anti-tumor drugs, and for studying the molecular mechanism of epithelial cancers [18]. In the two-stage skin carcinogenesis model, tumors are initiated by application of a single sub-carcinogenic dose of a carcinogen such as DMBA (a polycyclic aromatic hydrocarbon), which is the most widely used initiating agent among chemical initiators. During this process, DMBA induces mutations in the Hras and Kras genes [19]. This irreversible event is further promoted by the repeated application of a tumor promoting agent such as phorbol ester and TPA [18]. After appropriate exposure to DMBA and TPA, papilloma develop as a clonal outgrowth of the skin, and subsequently progress to invasive squamous cell carcinoma (SCC) at 20 weeks [20]. Various doses of the initiator and promoter have been applied to several mouse strains to develop the papilloma, although many studies used a specific dose of DMBA (25 μg) and TPA (4 μg) [21, 22]. Especially, BALB/c mice were exposed to two different doses (4 μg and 2 μg) of TPA; 4 μg TPA was observed to induce a higher incidence (84%) of papilloma in BALB/c mice, as compared to 2 μg TPA (17%) in the same strain [23, 24]. In the present study, three substrains of BALB/c mice were treated with DMBA (25 μg) and TPA (4 μg) to compare the skin tumor induction efficacy, histopathological structures, and expressions of tumor related proteins in response to DMBA+TPA cotreatment. This dose of TPA successfully induced skin tumors in all experimental groups, although few differences were observed during tumor development.

To determine whether strain background influences the efficacy of tumor induction, strain differences for two-stage chemical carcinogenesis have previously been evaluated in few studies. The outbred SENCAR mice showed high sensitivity to DMBA and TPA, while the inbred DBA/2 and C3H exhibited resistant effects for the two-stage protocol [25–27]. This protocol was also used to induce papilloma in 14 inbred, 2 hybrid, and 15 other genetic stocks. High level of induction efficacy (100–64%) was observed in the RHJ/Le, ABJ/Le, I/LnJ and BALB/cJ strains, whereas intermediate efficacy (58–42%) was detected in the BALB/cByJ, CBA/J and C57BL/6 J strains [24]. Furthermore, the susceptibility to two-stage skin carcinogenesis using DMBA and croton oil was evaluated in LACA and BALB/c mice. DMBA was found to be more sensitive in LACA mice than in BALB/c mice, although the metabolic activation of DMBA to the active carcinogen was not limited to the resistant strain [28]. In the current study, we investigated the influence of substrain backgrounds derived from three different sources, on the efficacy of skin tumor induction. Our results show the first evidence that BALB/cKorl, BALB/cA and BALB/cB substrains exhibit similar responses to two-stage skin carcinogenesis and therapeutic effect of cisplatin, in the DMBA+TPA treated model. However, the average number and induction efficacy of tumors were greater in BALB/cKorl substrain than in BALB/cA and BALB/cA. We though that this difference is attributable to the some unknown environmental stress during transportation and early breeding before purchase because the adaptation period and the experimental conditions are the same.

## Conclusion

We evaluated the influence of BALB/c substrain background on the DMBA+TPA-induced skin tumor development. Our results indicate that except few differences in the tumor induction rate, this two-stage chemical carcinogenesis model has overall similar functionality and reactivity to DMBA+TPA as well as to anti-cancer drugs, for all three BALB/c substrains evaluated. The results of the present study indicate that the BALB/cKorl mice and BALB/c mice from other commercial suppliers can be widely applied to produce a carcinogenesis model through treatment of carcinogen.

## Data Availability

Available.

## Abbreviations

Cisplatin: Cis-diamine dichloroplatinum (II), Pt (NH3)2Cl2
DMBA: 7,12-Dimethylbenz[a]anthracene
TPA: 12-O-Tetradecanoyl Phorbol-13-Acetate
