# Correction to: Influence of three BALB/c substrain backgrounds on the skin tumor induction efficacy to DMBA and TPA cotreatment

**DOI:** 10.1186/s42826-020-00067-9

**Published:** 2020-10-06

**Authors:** Mi Ju Kang, Jeong Eun Gong, Ji Eun Kim, Hyeon Jun Choi, Su Ji Bae, Yun Ju Choi, Su Jin Lee, Min-Soo Seo, Kil Soo Kim, Young-Suk Jung, Joon-Yong Cho, Yong Lim, Dae Youn Hwang

**Affiliations:** 1 Department of Biomaterials Science, College of Natural Resources and Life Science/Life and Industry Convergence Research Institute/Laboratory Animals Resources Center, Pusan National University, Miryang, South Korea; 2 Laboratory Animal Center, Daegu-Gyeongbuk Medical Innovation Foundation, Daegu, South Korea; 3 College of Veterinary Medicine, Kyungpook National University, Daegu, South Korea; 4 College of Pharmacy, Pusan National University, Busan, South Korea; 5 Exercise Biochemistry Laboratory, Korea National Sport University, Seoul, South Korea; 6 Department of Clinical Laboratory Science, College of Nursing and Healthcare Science, Dong-Eui University, Busan, South Korea

**Correction to: Lab Anim Res 36, 30 (2020)**

**https://doi.org/10.1186/s42826-020-00063-z**

It was highlighted that the in original article [1] part of Fig. 1 was missing. This Correction article shows the correct Fig. 1. The original article has been updated.

**Figure 1:**
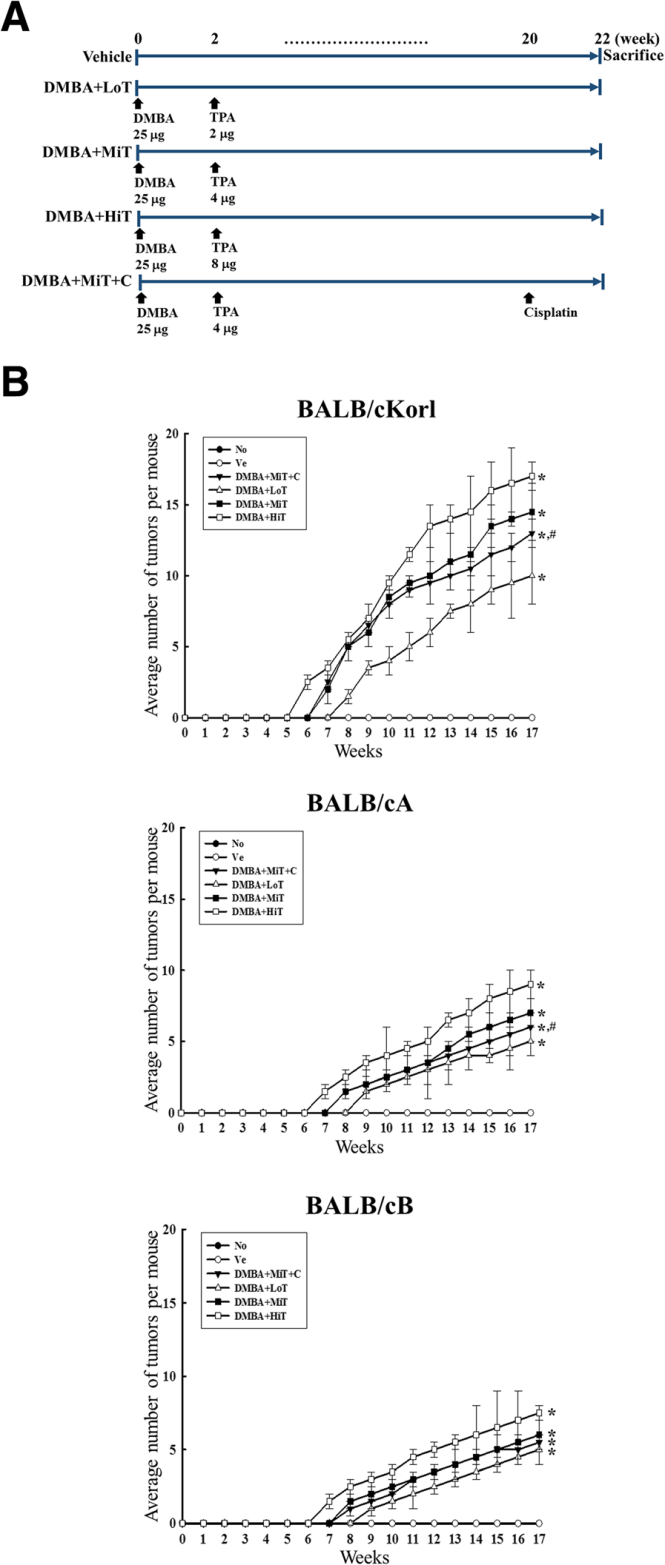
Fig. 1
